# Prediction of Potential Suitable Areas and Priority Protection for *Cupressus gigantea* on the Tibetan Plateau

**DOI:** 10.3390/plants13060896

**Published:** 2024-03-20

**Authors:** Huayong Zhang, Yanan Wei, Junjie Yue, Zhongyu Wang, Hengchao Zou, Xiande Ji, Shijia Zhang, Zhao Liu

**Affiliations:** 1 Research Center for Engineering Ecology and Nonlinear Science, North China Electric Power University, Beijing 102206, Chinazouhc@ncepu.edu.cn (H.Z.); 2 Theoretical Ecology and Engineering Ecology Research Group, School of Life Sciences, Shandong University, Qingdao 250100, China; 3 Energy Conversion Group, Energy and Sustainability Research Institute Groningen, Faculty of Science and Engineering, University of Groningen, Nijenborgh 6, 9747 AG Groningen, The Netherlands; 4 Research Group WILD Department Biology, Vrije Universiteit Brussel, Pleinlaan 2, 1050 Brussels, Belgium

**Keywords:** Biomod2, Marxan, climate change, environmental variables, species distribution models

## Abstract

*Cupressus gigantea* (*C. gigantea*) is an endemic endangered species on the Tibetan Plateau; its potential suitable areas and priority protection in the context of global climate change remain poorly predicted. This study utilized Biomod2 and Marxan to assess the potential suitable areas and priority protection for *C. gigantea*. Our study revealed that the suitable areas of *C. gigantea* were concentrated in the southeastern Tibetan Plateau, with the center in Lang County. Temperature was identified as a crucial environmental factor influencing the distribution of *C. gigantea*. Over the coming decades, the suitable range of *C. gigantea* expanded modestly, while its overall distribution remained relatively stable. Moreover, the center of the highly suitable areas tended to migrate towards Milin County in the northeast. Presently, significant areas for improvement are needed to establish protected areas for *C. gigantea*. The most feasible priority protected areas were located between the Lang and Milin counties in Tibet, which have more concentrated and undisturbed habitats. These results provide scientific guidance for the conservation and planning of *C. gigantea*, contributing to the stability and sustainability of ecosystems.

## 1. Introduction

The Tibetan Plateau, known as the “third pole” of the Earth, is a distinct geomorphological unit [1,2] where the temperature has risen substantially over the past few years, rendering it one of the most rapidly warming regions worldwide. Vegetation distribution on the plateau has thus been profoundly affected by climate change [3,4]. In the context of global climate change, some endangered species are gradually expanding as a result of successful adaptation to the changing environment [5,6,7], while others are declining further due to poor adaptation [8,9]. Variations in vegetation distribution induced by climate change pose a significant challenge to the ecosystem’s stability and the conservation of endangered species on the Tibetan Plateau [10,11]. Therefore, exploring the responses of endangered species to climate change is essential to safeguard and protect local ecological equilibrium and biodiversity.

*Cupressus gigantea* W. C. Cheng & L. K. Fu (*C. gigantea*) is a member of the family *Cypressaceae*, a genus of *Cupressus* Linn. and is narrowly restricted to the dry valleys of the Nyang River and the Brahmaputra River on the Tibetan Plateau with an elevation of 3000–4000 m [12,13]. It is an endemic species with long geological history on the Tibetan Plateau, holding great significance in the research of cypress phylogeny, vegetation development, and its environmental relationships [14,15,16,17]. Meanwhile, the *C. gigantea*, with its tall trunk, distinctive fragrance, and high quality wood [14,16], plays an important role in Tibetan incense production, urban greening, and timber production, and has high ecological and economic values [15,18,19]. However, the *C. gigantea* has a low seed-setting rate, hindering natural expansion [20]. Extensive logging for Tibetan incense production and timber utilization have further contributed to its decline [18,19]. Nowadays, *C. gigantea* faces extinction with a fragmented and limited distribution [16,21] and has been listed as endangered in the Red List of Chinese Species. Despite extensive research on the phylogeny and cultivation of *C. gigantea*, there is a notable lack of studies on its potential suitable areas and the establishment of protected areas for its conservation.

Evaluating potential suitable areas and establishing priority protected areas are crucial strategies for the conservation of endangered species [22,23]. Owing to a high precision and accuracy, the ensemble model (EM) in Biomod2 is widely employed to predict potential suitable areas for species distribution and identify the environmental drivers behind the selection of such areas [24,25,26]. Previous studies have applied this model to accurately predict the distribution of various vegetation types, such as grasslands and forests, under the influence of climate change [27,28]. Furthermore, the Marxan software version 2.4.3 is a popular conservation planning software that has been widely used in system protection planning and exhibits excellent performance in identifying priority protected areas [29,30,31]. Marxan operates on the principle of complementarity, utilizing the annealing algorithm to iteratively identify the optimal set of planning units through repeated screening [32,33]. Unlike the Biomod2 modeling platform, which focuses on species distributions based on natural environmental variables (e.g., temperature, precipitation, and soil), Marxan considers socio-economic factors and aims to identify priority protected areas that reduce human–ecological conflicts [22,30]. Therefore, combining Biomod2 and Marxan can contribute to the conservation and management of endangered species by more efficiently identifying potential suitable areas and priority protected areas.

In this study, we evaluated the distribution of *C. gigantea* under three climate scenarios (SSP126, SSP370, and SSP585) for the current and future climate (2041–2060 and 2061–2080), respectively. Our objectives are as follows: (1) using Biomod2 modeling to predict the potential suitable areas and migration trends of *C. gigantea* under current and future scenarios, and to explore the key influencing factors of potential suitable areas; (2) combining superposition analysis with Marxan to determine the potential suitable areas and priority protected areas for the *C. gigantea*. This study examines the alterations in the potential suitable areas of *C. gigantea* in response to global environmental changes. It offers valuable recommendations for establishing priority protected areas dedicated to *C. gigantea*. Our findings contribute significantly to the ecological restoration of endangered species, as well as the promotion of ecosystem stability and biodiversity conservation.

## 2. Results

### 2.1. Optimal Model and Accuracy Evaluation

Based on the C. gigantea base distribution data and environmental data, all ten selected single models accurately predicted their fitness zones (Table 1). XGBOOST and RF demonstrated the highest performance (AUC > 0.99, TSS > 0.99), indicating exceptional accuracy and stability. GBM, GLM, CTA, FDA, MARE, MAXENT, and ANN also showed good performance (TSS > 0.9, AUC > 0.9). However, the predictive ability of the SRE model was relatively modest, with TSS and AUC values averaging 0.75 and 0.88, respectively. In this study, post-modeling analyses were carried out using an EM. The TSS and AUC of the EM were calculated, revealing that the combined model improved accuracy to a certain extent, with TSS and AUC values of 0.999.

### 2.2. Current Potential Suitable Areas and Environmental Drivers

**Figure 1:**
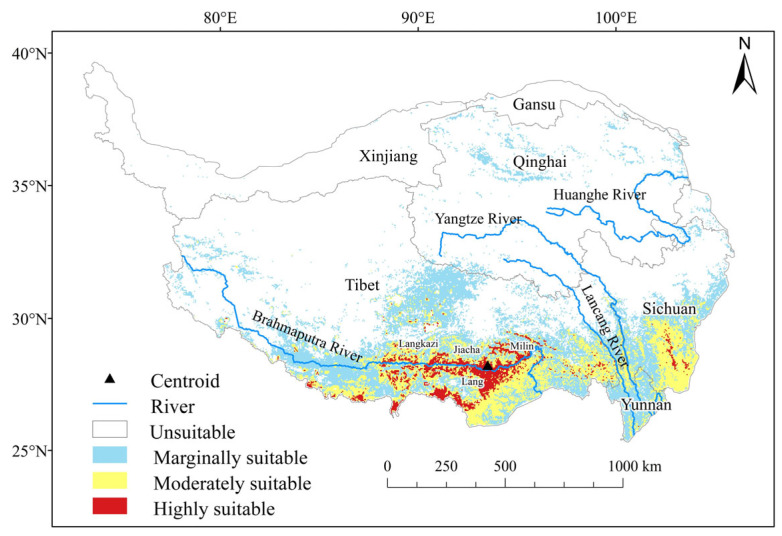
Potential suitable areas of *Cupressus gigantea* W. C. Cheng & L. K. Fu under current climate conditions.

The predictions generated by the EM indicate that current climatic conditions support the distribution of *C. gigantea* in the southern region of the Tibetan Plateau, with sporadic occurrences in the northeastern part, accounting for approximately 24.28% of the total area of the Tibetan Plateau (Figure 1). The marginally and moderately suitable areas comprise the majority of the suitable area of *C. gigantea*. The highly suitable areas are limited and primarily concentrated downstream of the Brahmaputra River, with the distribution center situated in Lang County, representing only 1.96% of the Tibetan Plateau.

The distribution pattern of *C. gigantea* is primarily influenced by climate, followed by soil texture, while terrain is less significant (Table 2). The results showed significant contributions from TSN (32.3%), ART (24.3%), PDQ (9.8%), and SAND (16.0%). MAP, CEC, SOC, and NITROGEN had a weak influence, while the remaining variables had insignificant effects. Overall, temperature was the main influencing factor on the distribution of *C. gigantea*, with a cumulative contribution of 56.6%.

### 2.3. Future Contraction and Expansion of Potential Suitable Areas

**Figure 2:**
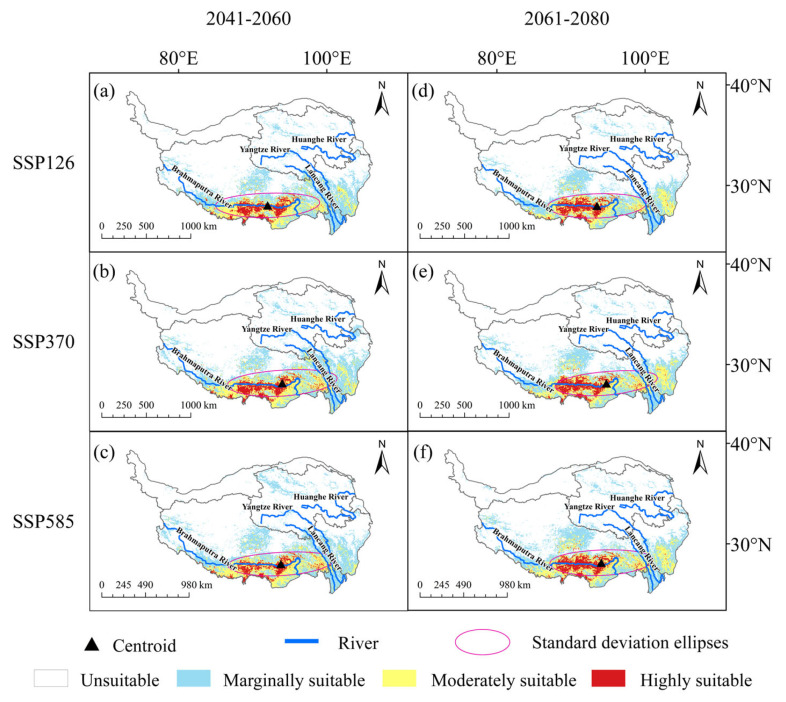
Potential suitable areas of *Cupressus gigantea* W. C. Cheng & L. K. Fu. (**a**) 2041–2060: SSP126; (**b**) 2041–2060: SSP370; (**c**) 2041–2060: SSP585; (**d**) 2061–2080: SSP126; (**e**) 2061–2080: SSP370; (**f**) 2061–2080: SSP126.

**Figure 3:**
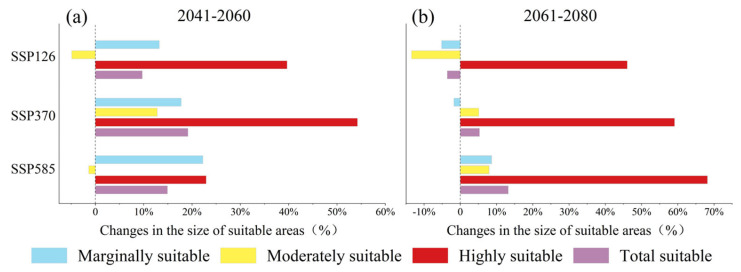
Changes in the potential suitable areas of *Cupressus gigantea* W. C. Cheng & L. K. Fu. (**a**) 2041–2060; (**b**) 2061–2080.

**Figure 4:**
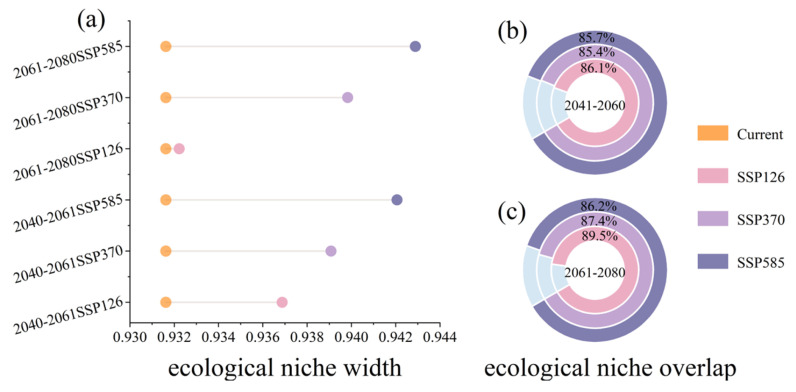
Ecological niche width and ecological niche overlap. (**a**) Ecological niche width; (**b**) ecological niche overlap: 2041–2060; (**c**) ecological niche overlap: 2061–2080.

Under future climate change scenarios, the potential suitable areas of *C. gigantea* were projected to expand. From 2041 to 2060, both the SSP126 and SSP580 scenarios showed similar patterns in the changes of potential suitable areas. Specifically, there was a significant expansion in the marginally and highly suitable areas, while the moderately suitable areas showed a slight contraction. In general, the potential suitable areas tended to expand, with expansion rates of 9.7% and 14.9%, respectively (Figure 2a,c and Figure 3a). In Scenario SSP370, all habitats showed an increasing trend, with the highest increasing rate of 54.2% for the highly suitable areas (Figure 2b and Figure 4a). From 2061 to 2080, the SSP126 scenarios predominantly indicated a contraction in the marginally and moderately suitable areas of *C. gigantea*, while the highly suitable areas expanded. The overall suitable areas experienced a slight contraction of 3.6%, making it the only scenario to exhibit a contraction trend (Figure 2d and Figure 3b). In the SSP370 scenario, the marginally and highly suitable areas expanded primarily, while the moderately suitable areas showed a slight contraction (Figure 2e and Figure 3b). Under the SSP585 scenario, all suitable areas expanded, with a total increasing rate of 13.2% (Figure 2f and Figure 3b).

Under the influence of climate change, the distribution range of *C. gigantea* exhibited an east–west pattern and a tendency to shift towards higher latitudes (Figure S1). However, the extent of migration was relatively tiny. Apart from the SSP126 (2041–2060) scenario, the center of mass for all the other scenarios was situated in the Lang and Milin counties, indicating a migration trend towards the northeast. Of these scenarios, the SSP126 (2061–2080) scenario exhibited the most minor extent of migration, which largely overlapped with the current center of mass, whereas the SSP370 (2061–2080) scenario showed the most considerable extent of migration.

The ecological niche of the *C. gigantea* remained relatively stable. The width of the ecological niche was projected to expand from 0.932 to 0.943 with increased carbon emissions, indicating a minimal overall change (Figure 4a). *C. gigantea* exhibited a relatively considerable niche overlap across different climatic scenarios, with the maximum overlap observed at 89.5% and the minimum overlap at 85.4% (Figure 4b,c), indicating no significant niche differentiation.

### 2.4. Priority Protected Areas

**Figure 5:**
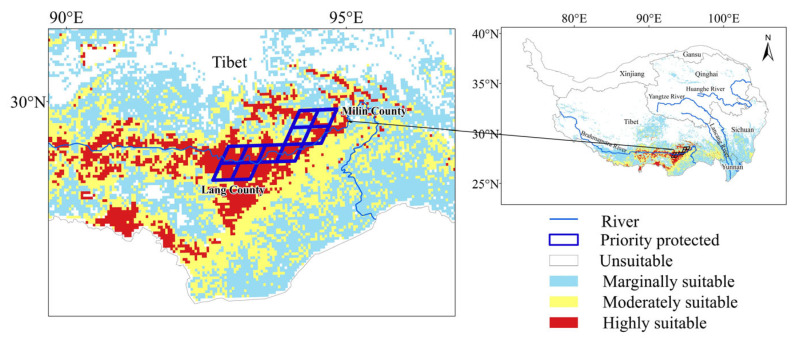
Stable potential suitable areas and priority protected areas.

The suitability zones derived from overlaying the potential suitable areas based on various climatic scenarios are characterized by increased stability and are highly conducive to cultivation. The fuzzy overlay analyses showed that the stable potential suitable areas of *C. gigantea* were relatively small, mainly situated in the southern Tibetan Plateau, encompassing only 20.44% of the total area. The stable highly suitable areas near Lang County cover a mere 1.49% of the Tibetan Plateau. The priority protected areas selected using the optimal Marxan strategy exhibit a higher concentration and lower levels of human disturbance. These priority protected areas are primarily situated between the Milin and Lang counties, encompassing 9000 km^2^ (Figure 5).

## 3. Discussion

This study incorporated environmental variables, including climate, soil, and terrain factors, and employed the EM in Biomod2 to investigate the potential suitable areas and environmental drivers of *C. gigantea* (Table 1). Correcting each other between single models effectively improves the stability of the prediction and model accuracy [28,34]. The model exhibited excellent overall performance (AUC > 0.99, TSS > 0.99) and accurately predicted the driving forces influencing the distribution of potential suitable areas for *C. gigantea*.

This study revealed that the potential suitable areas of *C. gigantea* are mainly located in the river terraces and valleys along the Brahmaputra River, with fewer highly suitable areas. This statement is consistent with previous studies on the main concentration of *C. gigantea* [12,35]. The limited extent of its highly suitable areas could be attributed to the distinctive environmental conditions of the Tibetan Plateau. Previous studies have shown that due to the unique climatic conditions of the Tibetan Plateau, the vegetation distribution is dominated by grasslands and deserts, with fewer forests [36,37]. In the southeastern Tibetan Plateau, the *C. gigantea* grows at an altitude of 3000 to 4000 m, the average annual temperature is around 8 °C, and the soil has a high sand content. *C. gigantea* has survived in the region for a long time and has adapted well to the growing environment [38].

As an endangered species endemic to the Tibetan Plateau, *C. gigantea* is affected by the severe environmental conditions of this region, which in turn influence its distribution [35,39]. Plants growing on the Tibetan Plateau, characterized by its high altitude and unique terrain, must adapt to extreme climatic events, including droughts and low temperatures. Temperature and precipitation are key factors influencing plateau vegetation distribution [40,41,42,43]. Previous studies have demonstrated that temperature plays a significant role in influencing the growth of *C. gigantea*, affecting its growth rate, fruiting rate, and sapling survival, particularly under colder and more variable temperature conditions [35,39]. Our findings supported this notion, indicating that temperature was the primary determinant for selecting suitable areas of *C. gigantea*. Species distribution was more influenced by the temperature difference than the mean temperature. As TSN and ART increase, there is a gradual shift from highly suitable areas to marginally suitable areas and eventually to unsuitable areas (Figure S1). The observed temperature sensitivity aligns with that of other endangered species on the Tibetan plateau, such as *Fritillaria unibracteata*, *Paeonia decomposita*, and *Paeonia rotundiloba* [17,44]. Furthermore, our study revealed that the distribution of *C. gigantea* is significantly affected by temperature and by precipitation patterns, particularly the PDQ. Insufficient precipitation and low soil moisture during the dry season can impede tree growth [45]. Despite the high drought tolerance of *C. gigantea*, prolonged droughts can hinder its growth and compromise its ability to sustain vital activities by retaining water [12].

The distribution of *C. gigantea* is determined not only by climate but also by the soil sand content, which can be attributed to its historical presence in river valleys. Previous studies have demonstrated that wild *C. gigantea* grows in semi-arid river valleys along the central Brahmaputra River. The soils in these areas are characterized by high permeability, sandiness, and alkaline pH [12,35]. Over an extended period, *C. gigantea* has gradually adapted to the soil conditions in these areas [38], showing a preference for sandy soils, which contrasts with the preference of other plant species for clay soils [46,47]. Moreover, studies on artificial cultivation have demonstrated that sandy soils are conducive to rapid root growth, high rates of root formation, and the development of many new roots [35]. Our study supported these findings, indicating that climate plays a crucial role in the habitat selection of *C. gigantea* and the impact of soil sand content cannot be ignored.

The distribution pattern of vegetation on the Tibetan Plateau is changing due to global climate change. Several studies indicate that the climate of the Tibetan Plateau will experience significant warming during the periods of 2041–2060 and 2061–2080, and that a considerable amount of vegetation on the plateau will exhibit an inclination towards expansion [37,48,49]. The study reveals that *C. gigantea* possesses a broad ecological niche width and, consequently, exhibits a strong capacity to adapt to changes in climate conditions. The potential suitable areas of *C. gigantea* are expected to expand slightly in the coming decades, which is consistent with the distribution pattern of amaranth, stems, and other vegetation observed on the Tibetan Plateau [6,50]. In addition, increased carbon emissions due to anthropogenic impacts often influence climate change. Our study revealed that the suitable areas for *C. gigantea* expanded across most scenarios. The SSP370 scenario, characterized by moderate CO_2_ emissions, exhibited the most favorable conditions for significant expansion of the potential suitable areas while varying between the scenarios of low CO_2_ emissions (SSP126) and high CO_2_ emissions (SSP585). Previous studies have shown that low carbon emissions have a weaker effect on climate. In contrast, high carbon emissions lead to a more unstable climate, which may lead to climate extremes and ultimately affect the stability of vegetation habitats [51,52].

Unlike endangered species such as *Meconopsis punicea* Maxim and *Stipa purpurea* Griseb found on the Tibetan Plateau [6,8], *C. gigantea* do not exhibit significant migratory behavior in their habitats, resulting in a more stable overall distribution. The ability of vegetation to adapt often plays a significant role in shaping changes within its habitat [53,54]. Previous studies have shown that species with wider ecological niche widths benefit from increased resource availability and have a greater chance of survival in the face of complex climate change [55,56]. Our results supported the finding that *C. gigantea* possesses a wide ecological niche width and experiences significant ecological niche overlap under future climate change scenarios, and this suggests its remarkable adaptability to environmental conditions and the similarity between its current and projected ranges. This finding aligns with the predictions of the EM, which forecasts a slight northwestward expansion of *C. gigantea* habitat and an overall stable distribution in response to future climate change. We propose that the ecological niche characteristics of *C. gigantea* play a pivotal role in facilitating its migration in response to climate change. However, the *C. gigantea* has a limited range, constraining its ability to expand and migrate. With the onset of global warming, a substantial amount of vegetation exhibits a tendency to migrate to higher elevations and latitudes. Consequently, the potential for natural expansion of the *C. gigantea* may be restricted, highlighting the need for increased attention to this issue.

Despite the high adaptability of *C. gigantea* to various growing conditions, it still faces considerable growth challenges due to human-induced disturbances. Previous studies have demonstrated that extensive logging, driven by the high demand for Tibetan incense, poses a significant threat to the growth and distribution of *C. gigantea* [16,17]. The establishment of priority protected areas is vital for the preservation of *C. gigantea*. In Linzhi County, the local population has designated *C. gigantea* as a watershed forest, leading to favorable growth conditions [12,57]. However, more priority protected areas for *C. gigantea* on the Tibetan Plateau must be protected. Our study findings strongly supported the establishment of a priority reserve in the Lang and Milin counties area. This area is the primary habitat for wild *C. gigantea*, exhibiting a concentrated distribution that corresponds closely with our findings of highly suitable areas. Additionally, it is situated adjacent to the established Yarlung Zangbu Grand Canyon Nature Reserve (Figure S2). Given the present circumstances, expanding the existing reserve or establishing a nearby priority reserve would effectively enhance the protection of the *C. gigantea*.

Currently, the wild *C. gigantea* has a highly restricted distribution, and achieving its complete ecological restoration by establishing priority protected areas may take time and effort. Therefore, the implementation of artificial cultivation systems is a necessary complementary measure. The *C. gigantea* has been identified as a critical species for cultivation on the Tibetan Plateau [19], and extensive research has been conducted on its biology, ecology, and nursery techniques [14,17]. Previous studies have demonstrated that a stable ecological environment is conducive to the growth and cultivation of vegetation. The stable potential suitable areas, derived from overlaying climate scenarios from different periods, suggest that the southeastern region of the Tibetan Plateau offers environmentally stable and suitable areas for the *C. gigantea*, which are adapted for cultivation in the region. As our study focused solely on the environmental factors influencing the distribution of *C. gigantea*, without considering the species interactions within potential suitable areas, the specific selection of cultivation sites can be effectively guided by the distribution patterns of local species. We hope to protect the value and ecological benefits of the *C. gigantea* and promote its sustainable development through intensive protection and artificial cultivation.

## 4. Materials and Methods

### 4.1. Data Collection and Assembly

**Figure 6:**
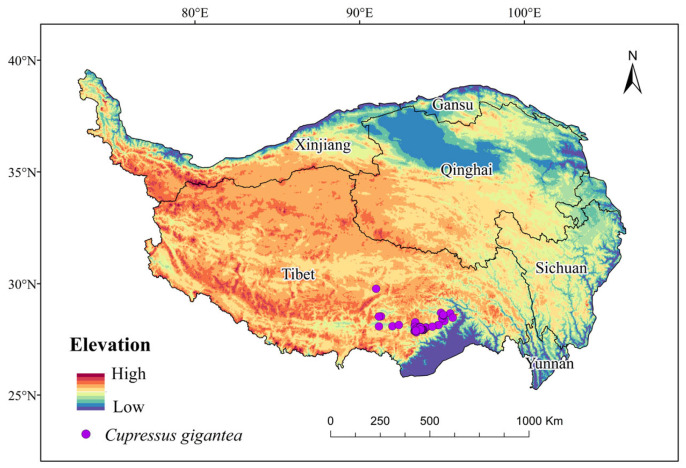
Distribution points of *Cupressus gigantea* W. C. Cheng & L. K. Fu.

The boundary data of the Tibetan Plateau were obtained from the National Tibetan Plateau Data Center (http://www.data.tpdc.ac.cn accessed on 5 June 2023). Species distribution data of *C. gigantea* were extracted from the Global Biodiversity Information Facility (GBIF, https://www.gbif.org/ accessed on 5 June 2023), the China Virtual Herbarium database (CVH, http://v5.cvh.org.cn/ accessed on 5 June 2023), the National Specimen Information Infrastructure (NSII, http://www.nsii.org.cn/ accessed on 5 June 2023), and the China Nature Digital Herbarium (CFH, http://cfh.ac.cn accessed on 5 June 2023); they were then integrated and compiled (Figure 6). ENMTools was employed to eliminate duplicate and closely located points to ensure modeling accuracy and avoid duplicate sampling.

The contemporary climate data were acquired from WorldClim version 2.1 (https://worldclim.org/; accessed on 5 June 2023) at a resolution of 0.25°, encompassing 19 bioclimatic variables. The soil data, consisting of 11 soil properties, were obtained from the global SoilGrids version 2.0 database (https://soilgrids.org; accessed on 5 December 2022) at a spatial resolution of 250 m. Terrain data (elevation) were obtained from WorldClim version 2.1 (https://worldclim.org/; accessed on 5 June 2023) at a spatial resolution of 0.25°. The human footprint (HFP) was acquired from the study [27], which was generated using eight human pressure variables (pasture, roads, railways, population density, navigable waterways, nighttime light, built environment, and cropland) at 1 km resolution.

The future climate data (19 bioclimatic variables) were acquired from WorldClim version 2.1 (https://worldclim.org/; accessed on 5 June 2023) at a resolution of 0.25°. The CMIP6 model represents a notable advancement over its predecessor, with findings indicating that it aligns more closely with future climate patterns and exhibits enhanced accuracy compared to CMIP5 projections [58,59,60]. Notably, simulation results for the Tibetan Plateau have shown significant improvement [59]. Among all GCMs, the BCC-CSM2-BR climate model developed by the Beijing Climate Centre (BCC) is widely recognized in China and has been extensively utilized for predicting both native and invasive species due to its high reliability [61,62,63]. In this study, we selected the BCC-CSM2-BR climate model from CMIP6 to examine the impact of different carbon emission scenarios. Specifically, we focused on three scenarios: SSP126, SSP370, and SSP585, which represent low to high carbon emissions. These scenarios were analyzed for two time periods: 2041–2060 and 2061–2080. The soil and terrain factors exhibit relative stability; thus, we set their parameters to remain constant under future climate conditions.

The spatial resolution was then standardized to 0.25° using ArcGIS 10.8 software, providing a basis for further research. Climate, terrain, and soil data were selected for Biomod2 analysis. We excluded variables with Pearson’s correlation coefficients (R > 0.8) to eliminate potential confounding effects. We ultimately retained five temperature variables, three precipitation variables, three soil texture variables, three soil fertility variables, and one terrain variable (Table S1). HFP data were selected as the foundational dataset for the Marxan software. The above data were assembled into a database for subsequent analysis.

### 4.2. Calculation of Potential Suitable Areas

Species distributions of *C. gigantea* were calculated for the current period and three future climate scenarios using the Biomod2 package [64] in R version 4.2.3 (http://www.r-project.org/ accessed on 5 June 2023). Initially, we selected ten individual models (RF, MaxEnt, FDA, GLM, GBM, CTA, ANN, MARS, XGBOOST, and SRE) in Biomod2 to predict the potential suitable areas. The accuracy was enhanced through inter-model correction, and a combined model was constructed using a weighted average for further analyses. Variations in the sample data can impact the model accuracy; we thus randomly divided the sample data (including occurrence data and pseudo-sampling points) into two sets: 75% of the data was assigned as the training dataset, while the remaining 25% served as the test dataset [65]. To ensure model stability, we performed 10 repetitions of the model algorithm for each sample dataset and calculated the average value. In addition, TSS and AUC were used as the evaluated criteria for the model accuracy (model is excellent when TSS > 0.9 and AUC > 0.9).

The potential suitable areas from EM were classified into four categories (highly suitable, moderately suitable, marginally suitable, and unsuitable areas) using the Natural Breaks method to assess contemporary and future distribution [66]. The expansion and contraction of *C. gigantea* potential suitable areas under future climate scenarios were then analyzed by comparing changes in the area of potential suitable areas. To evaluate the migration status of potential suitable areas, we employed the mean center method to calculate the centroid of the distribution of the highly suitable areas in each case. Subsequently, we conducted a standard deviation ellipse analysis to examine the distribution characteristics and movement direction. The long and short axes indicated the distribution range, while the deflection angle represented the distribution direction.

The species distribution is often influenced by ecological niche width and the degree of ecological niche overlap. We analyzed the changes in the ecological niche of *C. gigantea* under different climate scenarios using ENMtools [67]. To quantify the breadth of the ecological niche in its environmental space (B2env), we calculated the Levins’ B2 value, which ranges from 0 to 1 [68]. Smaller values indicate a narrower ecological niche with reduced adaptability, while larger values indicate a wider ecological niche with greater utilization of diverse resources and increased adaptability [69]. The overlap of ecological niches was quantified using Schoener’s D, which provides values ranging from 0 to 1 [67]. Greater values indicate a higher degree of ecological overlap and reduced variation in survival environments [70].

### 4.3. Recognition of Priority Protection

Using the fuzzy overlay technique, we conducted an overlay analysis of the current scenario and six future scenarios (2040–2061: SSP126, SSP370, SSP585; 2061–2080: SSP126, SSP370, SSP585) to identify stable potential suitable areas for establishing cultivated planting areas. To rationalize the establishment of priority protected areas, human footprint data were utilized as unit cost data for planning the establishment of protected areas. Multiple studies have consistently demonstrated that establishing protected areas in regions with a high human footprint index is more challenging and expensive [32,71]. Utilizing species distribution data and cost data, Marxan was employed to determine the priority protected areas for *C. gigantea*. The boundary length modifier (BLM) was set to 1, considering the dense distribution and limited fragmentation of *C. gigantea*. By the characteristics of the protected target, the preservation goal for the endangered *C. gigantea* was set at 80% [30]. The number of runs was set to 100, and other values were kept as default. Subsequently, the best option was selected, which entailed constructing a priority protected area with minimal protection costs and maximum spatial concentration.

## 5. Conclusions

Given the global climate change context, the ecosystems of the Tibetan Plateau are undergoing significant changes. Consequently, safeguarding endangered species becomes crucial for maintaining ecosystem stability and preserving biodiversity. In this study, based on the EM of Biomod2 and the Marxan software, we comprehensively analyzed the potential suitable areas and priority protected areas of *C. gigantea* by integrating species distribution data, climatic data, soil data, and topographic data. Our findings revealed that (1) temperature is the primary factor influencing the distribution of the *C. gigantea*, while soil and precipitation play a supportive role. The habitat of the *C. gigantea* is primarily concentrated in the southeastern Tibetan Plateau, with a relatively small, highly suitable area (1.96% of the plateau). Under the influence of climate change, the potential suitable areas are experiencing a slight expansion. Among different climate scenarios, the SSP370 scenario, characterized by moderate carbon emissions, is the most favorable for the growth of the *C. gigantea*. In contrast, the changes in potential suitable areas under the SSP126 and SSP585 scenarios fluctuate. (2) The potential suitable areas of the *C. gigantea* has shown a slight expansion towards the northeastern high-latitude areas. Due to the broad ecological niche of the thousand-headed cedar, its potential suitable areas under future climate conditions overlaps significantly with that of the present day, resulting in a small expansion of its potential suitable areas and a more stable overall distribution. (3) To ensure the survival of the *C. gigantea*, it is crucial to establish priority protected areas between the Milin and Lang counties. Additionally, adaptive cultivation areas should be established in the southeastern part of the Tibetan Plateau. By understanding the habitat of the *C. gigantea* and implementing priority protected measures, we can provide valuable scientific guidance for *C. gigantea* protection and planning and promoting sustainable development and biodiversity conservation.

## Figures and Tables

**Table 1:** AUC and TSS scores for the ten statistical modeling techniques used in this study.

Model	Evaluation Index		Model	Evaluation Index	
	AUC	TSS		AUC	TSS
XGBOOST	0.999	0.999	FDA	0.999	0.990
RF	0.999	0.999	MARS	0.997	0.975
GBM	0.999	0.996	MAXENT	0.980	0.942
GLM	0.999	0.992	ANN	0.956	0.900
CTA	0.976	0.922	SRE	0.881	0.749
EM	0.999	0.999			

**Table 2:** The importance of environment variables.

Category	Abbreviations	Environmental Variables	Important Value (%)
Temperature	MAT	Annual mean temperature	1.104
Precipitation	MDR	Mean diurnal range	1.313
	Iso	Isothermality	0.782
	TSN	Temperature seasonality	32.343
	ART	Temperature annual range	24.302
	MAP	Annual precipitation	2.127
	PDQ	Precipitation of driest quarter	9.858
	PCOQ	Precipitation of coldest quarter	0.709
Soil texture	SAND	Sand content (%)	16.047
	BDOD	Bulk density (kg/m^3^)	0.178
	CFVO	Coarse fragments volumetric (%)	0.646
Soil fertility	NITROGEN	Total nitrogen (g/kg)	1.603
	SOC	Soil organic carbon content (g/kg)	3.906
	CEC	Cation exchange capacity (cmolc/kg)	4.069
Terrain	ELE	Elevation(m)	0.388

## Data Availability

All links to input data are reported in the manuscript and all output data are available upon request to the authors.

## Supplementary Materials

The following supporting information can be downloaded at: https://www.mdpi.com/article/10.3390/plants13060896/s1, Figure S1. The centroid and standard deviation ellipses of highly suitable areas; Figure S2. Distribution of TSN and ART in different potential suitable areas; Table S1. All variables and their abbreviations used in our study.
