# Supplementary material for: Prediction of Potential Suitable Areas and Priority Protection for Cupressus gigantea on the Tibetan Plateau
Source: Plants (Basel). 2024 Mar 20;13(6):896. doi: 10.3390/plants13060896 (PMC10974514; doi:10.3390/plants13060896)
Supplement: Supplementary file 1 [file plants-13-00896-s001.zip › plants-2859972-supplementary.pdf]

Supplementary Information

# Prediction of Potential Suitable Areas and Priority Protection for *Cupressus gigantea* on the Tibetan Plateau

**Huayong Zhang [1,2,*], Yanan Wei [1], Junjie Yue [1], Zhongyu Wang [1], Hengchao Zou [1], Xiande Ji [3], Shijia Zhang [4] and Zhao Liu [2]**

[1] Research Center for Engineering Ecology and Nonlinear Science, North China Electric Power University, Beijing 102206, China; zouhc@ncepu.edu.cn (H.Z.)
[2] Theoretical Ecology and Engineering Ecology Research Group, School of Life Sciences, Shandong University, Qingdao 250100, China
[3] Energy Conversion Group, Energy and Sustainability Research Institute Groningen, Faculty of Science
and Engineering, University of Groningen, Nijenborgh 6, 9747AG Groningen, The Netherlands
[4] Research Group WILD Department Biology, Vrije Universiteit Brussel, Pleinlaan 2, 1050 Brussels, Belgium
* Correspondence: zhanghuayong@sdu.edu.cn

## This word file includes

Figure and Table Legends

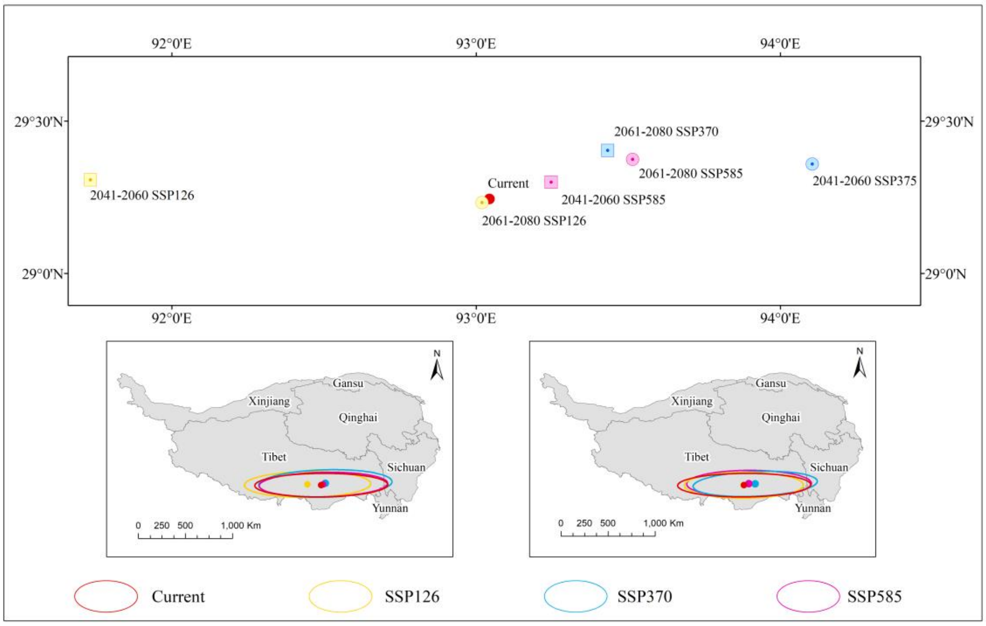


**Figure S1.** The centroid and standard deviation ellipses of highly suitable areas.

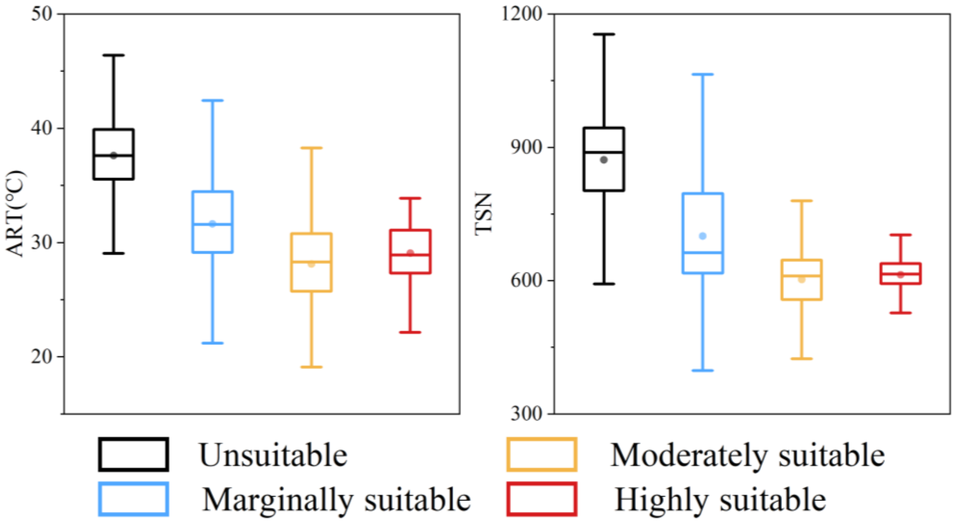


**Figure S2.** Distribution of TSN and ART in different potential suitable areas.

**Table S1.** All variables and their abbreviations used in our study.

| Groups | Abbreviations | Environmental variables | Source [resolution] |
|---|---|---|---|
| Temperature | MAT | Annual mean temperature | WorldClim version 2.1 [0.25°] |
| | MDR | Mean diurnal range | |
| | Iso | Isothemality | |
| | Tmax | Max temperature of warmest month | |
| | Tmin | Min temperature of coldest month | |
| | MTWEQ | Mean temperature of wettest quarter | |
| | MTDQ | Mean temperature of driest quarter | |
| | MTWQ | Mean temperature of warmest quarter | |
| | MTCQ | Mean temperature of coldest quarter | |
| | TSN | Temperature seasonality | |
| | ART | Temperature annual range | |
| Precipitation | MAP | Annual precipitation | |
| | PWM | Precipitation of wettest month | |
| | PDM | Precipitation of driest month | |
| | PWQ | Precipitation of wettest quarter | |
| | PDQ | Precipitation of driest quarter | |
| | PWAQ | Precipitation of warmest quarter | |
| | PCOQ | Precipitation of coldest quarter | |
| | PSN | Precipitation seasonality | |
| Soil texture | BDOD | Bulk density (kg/m$^3$) | SoilGrids 2.0[250m] |
| | CLAY | Clay content (%) | |
| | CFVO | Coarse fragments volumetric (%) | |
| | SILT | Silt content (%) | |
| | SAND | Sand content (%) | |
| Soil fertility | CEC | Cation exchange capacity (cmolc/kg) | |
| | OCD | Soil organic carbon density (kg/m$^3$) | |
| | OCS | Soil organic carbon stock | |
| | SOC | Soil organic carbon content (g/kg) | |
| | PHH2O | Soil pH x 10 in H2O | |
| | NITROGEN | Total nitrogen (g/kg) | |
| Terrain | ELE | Elevation(m) | WorldClim version 2.1 [0.25°] |
